# Novel maleic anhydride derivatives: liquid crystalline materials with enhanced mesomorphic and optical characteristics

**DOI:** 10.3389/fchem.2023.1287883

**Published:** 2023-11-09

**Authors:** Hoda A. Ahmed, Tariq Z. Abolibda, Yasser A. M. Ismail, Abdullah Almohammedi, K. A. Aly, Mohamed S. Ibrahim, Sobhi M. Gomha

**Affiliations:** ^1^ Department of Chemistry, Faculty of Science, Cairo University, Giza, Egypt; ^2^ Chemistry Department, College of Sciences, Taibah University, Yanbu, Saudi Arabia; ^3^ Department of Chemistry, Faculty of Science, Islamic University of Madinah, Madinah, Saudi Arabia; ^4^ Department of Physics, Faculty of Science, Islamic University of Madinah, Madinah, Saudi Arabia; ^5^ Department of Physics, Collage of Science and Arts Khylais, University of Jeddah, Khulais, Saudi Arabia; ^6^ Department of Physics, Faculty of Science, Al-Azhar University, Assiut Branch, Assiut, Egypt

**Keywords:** liquid crystal, maleic anhydride, N-arylmaleimide derivatives, nematic phase, transition entropy

## Abstract

A new class of liquid crystalline materials, 4-(2,5-dioxo-2,5-dihydro-1*H*-pyrrol-1-yl)phenyl 4-(alkoxy)benzoates (Mn), derived from maleic anhydride, was synthesized and studied for mesomorphic and optical properties. These materials consist of three derivatives with varying terminal flexible chain lengths (6–12 carbons) linked to the phenyl ring near the ester bond. The study employed differential scanning calorimetry and polarized optical microscopy (POM) to characterize the mesomorphic properties. Molecular structures were elucidated using elemental analysis, FT-IR, and NMR spectroscopy. The findings reveal that all the synthesized maleic anhydride derivatives exhibit enantiotropic nematic (N) mesophases. The insertion of the heterocyclic maleic anhydride moiety into the molecular structure influences the stability and range of the N phase. Additionally, entropy changes during N-isotropic transitions are of small magnitude and exhibit non-linear trends independent of the terminal alkoxy chain length (n). This suggests that the ester linkage group does not significantly promote molecular biaxiality, and the clearing temperature values are relatively high. By comparing the investigated materials with their furan derivatives found in existing literature, it was established that the substitution examined in this study induces the formation of nematic phases.

## Graphical Abstract

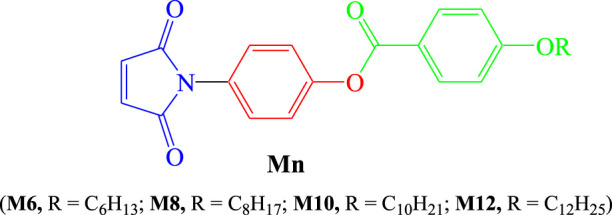

## 1 Introduction

Today, all types of display devices, including computer and laptop displays, TVs, clocks, visors, and navigational systems, use liquid crystals. When a substance is in a liquid crystal state, it possesses characteristics of both liquids and crystals. Their novel optical, electrical, and mechanical features have garnered a lot of interest. Each liquid crystal assembly that makes up a display pixel is governed by its own electromagnetic field. The field modifies the liquid crystals’ orientation, which alters how much light can pass through them and result in the images you see on a screen (Alamro et al., 2021; Gomha et al., 2021; Abdou et al., 2022; Alamro et al., 2022; Chi et al., 2022; Uchida et al., 2022; Wang et al., 2022; Yin et al., 2022; Behera et al., 2023; Ma et al., 2023).

The greater coast and lower conversion efficiency of solar energy now restrict its application. One of the most crucial areas to investigate for cutting costs and boosting conversion efficiencies in solar systems is the performance of several basic organic derivatives (Zhang et al., 2018; Li et al., 2019). Solar energy applications such catalytic photodegradation of dyes, solar hydrogen generation, photo-electrochemical water splitting, and solar cells rely heavily on band gap engineering and optical property management (Ahmed et al., 2020).

In recent times, maleimides have gained recognition as significant pharmacophores and have assumed a crucial role as medicinal agents, exhibiting diverse biological activities. These activities encompass antibacterial properties (López et al., 2003), analgesic effects (Mahle et al., 2010), antistress attributes (Badru et al., 2012), antiprotozoal capabilities (Dürüst et al., 2012), antiangiogenic functions (Acero et al., 2012), as well as cytotoxicity, DNA binding, and apoptotic inducing activity (Alaa, 2007a). Various derivatives of maleimides have been identified as selective inhibitors of specific enzymes, including monoglyceride lipase (Matuszak et al., 2009), GSK-3a (Sivaprakasam et al., 2006), Cdc25B (Chen et al., 2010), Bfl-1 (JCashman et al., 2010), and DNMT-1 (Suzuki et al., 2010). The synthesis and biological evaluation of N-aryl maleimide analogs have been extensively explored. N-aryl maleimides serve as the structural framework for numerous natural products such as polycitrin (Burtoloso et al., 2006) and camphorataimides (Stewart et al., 2007). Maleimide and its derivatives are synthesized from maleic anhydride through a process involving treatment with amines followed by dehydration (Birkinshaw et al., 1963). Maleimide derivatives exhibit significant appeal in terms of their chemical reactivity. They participate in fascinating reactions like Diels–Alder reactions with dienes and nucleophilic Michael-type additions of thiols or amines to the vinylene moiety. The unsaturated imide serves as a crucial building block in organic synthesis. Moreover, maleimide encompasses a group of derivatives derived from the potent maleimide, wherein the NH group is substituted with alkyl or aryl groups such as methyl or phenyl, respectively. Additionally, the vinylene group within the maleimide moiety, featuring a 1,2-disubstituted ethylene structure, can undergo polymerization with radical or anionic initiators. This process yields a polymer with exceptional thermostability or heat-resisting properties, which can be further copolymerized with vinyl acetate (A et al., 2014). The addition of a fused-heterocycle moiety to the molecular structure has been thoroughly examined in the quest to create new mesogenic cores, and the results show a variety of novel mesomorphic features (Han, 2013; Ghosh and Lehmann, 2017). In addition to increasing the species of liquid crystals, the addition of heteroatoms like nitrogen, sulphur, and oxygen also has a significant impact on the thermal and geometrical properties of the materials under investigation (Seed, 2007; Ha et al., 2010; Tariq et al., 2013; Merlo et al., 2018; Yeap et al., 2018; Weng et al., 2019a; Weng et al., 2019b; Ren et al., 2019). Insertion of fused-aromatic rings into a central or terminal structural shape alters the dielectric constant, polarizability, geometry, and mesophase transition temperatures (Seed, 2007; Ha et al., 2010; Tariq et al., 2013; Merlo et al., 2018; Yeap et al., 2018; Weng et al., 2019a; Weng et al., 2019b; Ren et al., 2019; Nafee et al., 2020a). Compounds contain imide groups, whether small molecules or macromolecules, display impressive electrical properties, favorable solubility in polar substances, resistance to hydrolysis, and high thermal stability (Bharel et al., 1993; Iijima et al., 1995; Langmuir et al., 1995; Hamper et al., 1996; Ohkubo et al., 1996; Bojarski et al., 2004; Alaa, 2007b; Mohammed and Mustapha, 2010a). These exceptional characteristics have prompted significant efforts to develop various imide-containing compounds composed of two carbonyl groups bonded to a nitrogen atom. The conventional method for synthesizing cyclic imides without substitutions involves heating dicarboxylic acids or their anhydrides with reactants such as ammonia, urea, formamide, lithium nitride, or primary amines (Handley et al., 1960; Gordon and Ehrenkaufer, 1971; Połoński et al., 2000; Wu et al., 2003). However, this reaction necessitates high temperatures to ensure efficient ring closure.

New classes of materials, liquid crystalline thermosets (LCTs) bring together the best qualities of thermotropic LC polymers and traditional thermosets. The network structures of these materials are made up of rigid-rod or extended chain segments that are cross-linked in three dimensions. Increased processability, higher glass transition temperatures, greater dielectric strength, and less shrinkage during curing are only a few of the benefits of LCTs (Lee et al., 2002; Jang and Bae, 2005; Cho et al., 2006). Additionally, they exhibit noteworthy qualities such as extremely low dielectric constants and dissipation factors, exceptional performance at high temperatures, chemical resistance, inherent flame retardance (Lee, 2006; Wang and Jiang, 2006), and low coefficients of thermal expansion (CTEs). These unique properties make LCTs highly desirable for advanced electronic applications. Moreover, efforts have been made to reduce the CTEs of LCT films in order to mitigate thermal stress in electronic laminates used in microelectronic applications (Hasegawa and Tominaga, 2005; Hasegawa et al., 2006; Morita et al., 2006). Functionalized rigid-rod oligomers or monomers are cross-linked in the mesophase to form either an isotropic or anisotropic network structure; these materials are known as thermotropic liquid crystals (LCs). Some thermotropic LCTs have been reported to possess cross-linkable units as end groups. Various functional units have been utilized as such end groups, including maleimide (Hoyt et al., 1990a), nadimide (Hoyt et al., 1990b), epoxy (Domszy and Shannon, 1990; Mallon and Adams, 1993; Carfagna et al., 1994a; Carfagna et al., 1994b; Carfagna et al., 1997), isocyanate (Mormann and Zimmermann, 1995), and acetylene (Langlois et al., 1994; Benicewicz et al., 1997; Gavri et al., 2001). Due to the importance of intermolecular interactions between mesogens in formation the liquid crystal phase, this characteristic is essential for the production of ordered structures in liquid crystals. Additionally, the aromatic rings also engage in - stacking interactions, which further improve the mesogen’s ordering capacity.

The purpose of this research, which is a continuation of our earlier work, is to create new derivatives of the terminal heterocyclic moiety, specifically 4-(2,5-dioxo-2,5-dihydro-1*H*-pyrrol-1-yl)phenyl 4-(alkoxy)benzoates, **Mn**. An ester linkage with a phenyl ring connected to various lengths of alkoxy groups and the other terminal is the maleic anhydride moiety. The project also aims to examine the mesomorphic and optical characteristics of the current system and investigate the impact of changing the flexible chain’s terminal length on their mesomorphic interaction.

## 2 Results and discussion

### 2.1 Synthesis

**SCHEME 1:**
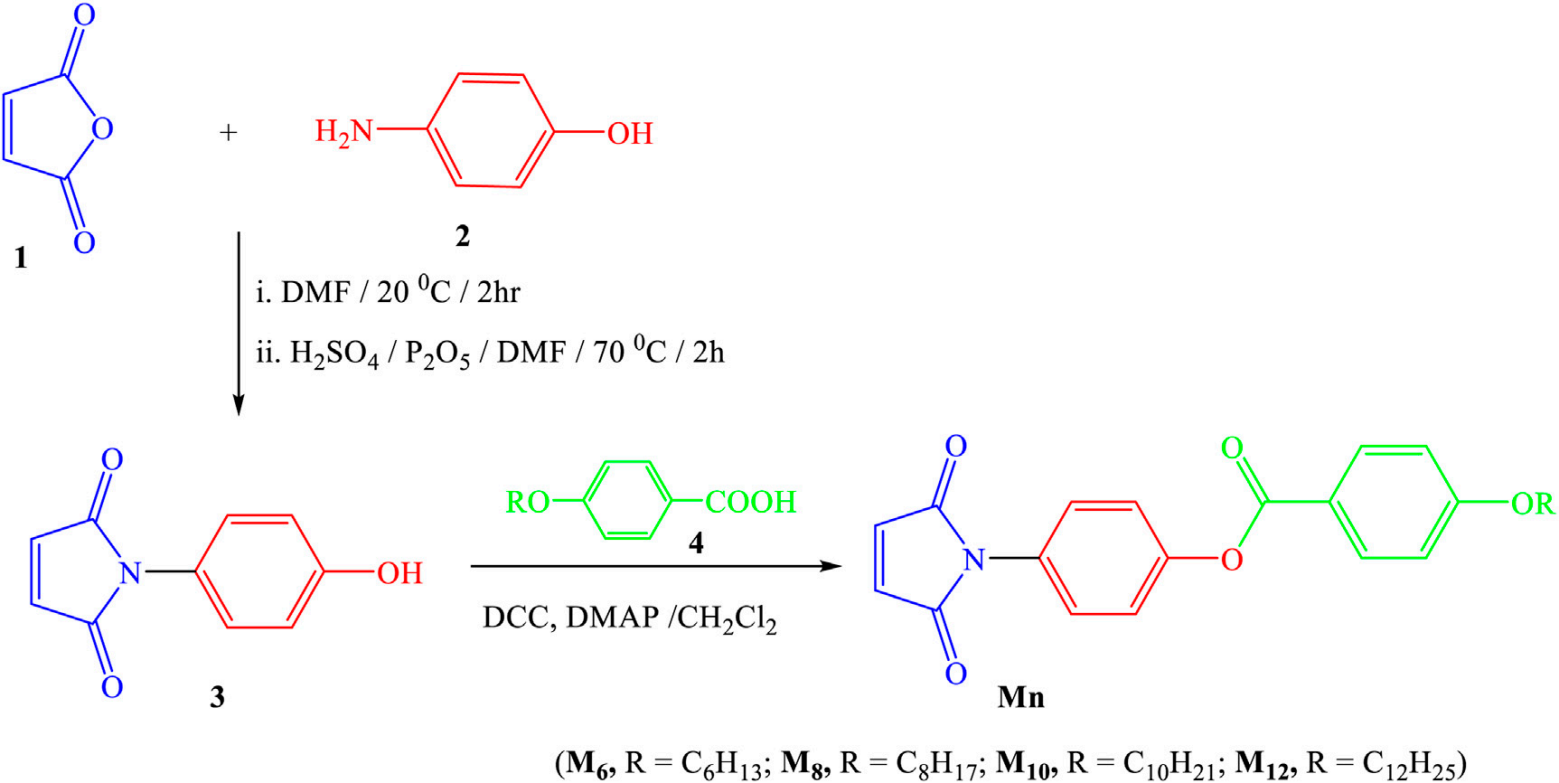
Synthesis route of title compounds **Mn**.

The process used to create the titled **Mn** compounds involves two sequential steps. It begins with synthesizing 1-(4-hydroxyphenyl)-1*H*-pyrrole-2,5-dione **3** by combining 4-hydroxyaniline **2** with maleic anhydride **1** as previously reported (Mohammed and Mustapha, 2010a). Next, compound **3** is transformed into the desired 4-(2,5-dioxo-2,5-dihydro-1*H*-pyrrol-1-yl)phenyl 4-(alkoxy)benzoates by reacting it with 4-alkoxybenzoic acid **4** in dry methylene chloride, along with N,N′-dicyclohexylcarbodiimide (DCC) and catalytic amounts of 4-dimethylaminopyridine (DMAP) (Scheme 1). The structure of compounds **Mn** was confirmed using elemental analyses and spectral data, including IR, ^1^H NMR, ^13^C NMR and MS.

### 2.2 Spectroscopic analysis

The ^1^H-NMR data provided essential structural information about the synthesized compounds **Mn**, confirming the successful synthesis of the intended product and the presence of characteristic functional groups. The ^1^H-NMR spectrum (300 MHz, CDCl_3_) of compound **M6** exhibited distinct peaks at δ/ppm values. The signals at 0.73–0.80 ppm were attributed to a triplet (t) representing the presence of three equivalent methyl protons (CH_3_). Additionally, a multiplet (m) pattern between 1.22 and 1.43 ppm indicated six protons arising from the aliphatic chain (CH_3_(CH_2_)_3_CH_2_CH_2_O-). Two more protons of this chain resonated as a multiplet in the range of 1.70–1.76 ppm. A triplet at 3.79–4.04 ppm corresponded to two protons associated with the methylene group of the aliphatic chain (CH_3_(CH_2_)_3_CH_2_CH_2_O-). In the aromatic region, three distinct doublets (d) were observed at 7.15, 7.24, and 7.37 ppm, each representing two protons of the aromatic ring. Additionally, a sharp singlet (s) appeared at 7.74 ppm, indicating the presence of two protons from the pyrrole moiety. Finally, another doublet at 8.12 ppm accounted for two protons of the aromatic ring.

Moreover, the ^13^C-NMR data provided valuable insights into the structural features of the unknown compounds, confirming the successful synthesis of the desired molecule and supporting its potential application in various biological and chemical studies. The ^13^C-NMR spectrum (75 MHz, CDCl_3_) of compound **M6** exhibited distinct peaks at various chemical shifts. The signal at 14.0 ppm were assigned to the methyl (CH_3_) group, while the peaks at 23.8, 25.6, 29.57, 31.0, and 68.2 ppm corresponded to the methylene (CH_2_) groups in the molecule. The resonances observed at 116.2, 121.5, 123.0, 127.9, 130.2, 131.5, 136.0, and 147.5 ppm were attributed to the aromatic carbon atoms (Ar-C) and the carbon-nitrogen (C=N) groups present in the structure. Additionally, signals at 161.6 ppm indicated the presence of conjugated double bonds involving aromatic carbons and C=N. Moreover, the signals at 163.1 and 166.3 ppm were attributed to the two carbonyl (C=O) carbon atoms.

Additionally, the infrared spectrum (IR) spectral data provide valuable insights into the molecular structure and functional groups present in the compound, aiding in its characterization and potential application in various fields. The infrared spectrum of compound **M6** recorded in KBr disc displayed characteristic vibrational bands at v3041 cm^-1^, corresponding to (=C-H) stretching vibrations, and at 2928 cm^-1^, attributed to (-C-H) stretching vibrations. Additionally, two prominent peaks were observed at 1686 cm^-1^ and 1729 cm^-1^, indicating the presence of two carbonyl groups (2 C=O) within the molecule. Furthermore, a peak at 1605 cm^-1^ was observed, which can be attributed to the stretching vibration of the carbon-nitrogen double bond (C=N), while a band at 1571 cm^-1^ signifies the presence of carbon-carbon double bonds (C=C).

Finally, the MS data provides valuable insights into the fragmentation pattern and molecular structure of compound **Mn**, aiding in its characterization and identification for further studies and potential applications. The results of the mass spectrometry (MS) analysis of compound **M6** revealed a molecular ion peak at m/z 393, corresponding to the intact molecular weight of the compound (M^+^). Another significant peak at m/z 307 was observed with 100% relative intensity, likely resulting from the loss of the hexyl group (C_6_H_13_) from the parent molecule. Moreover, the peak at m/z 292 (71%) suggests the further fragmentation of the compound. Other notable peaks included m/z 231 (63%) indicating possible fragmentation involving the phenyl pyrrole moiety, m/z 189 (38%) suggesting the loss of both the hexyl and phenyl pyrrole moieties, and m/z 162 (81%) representing a common fragment found in the MS analysis.

### 2.3 Mesomorphic examinations

**FIGURE 1:**
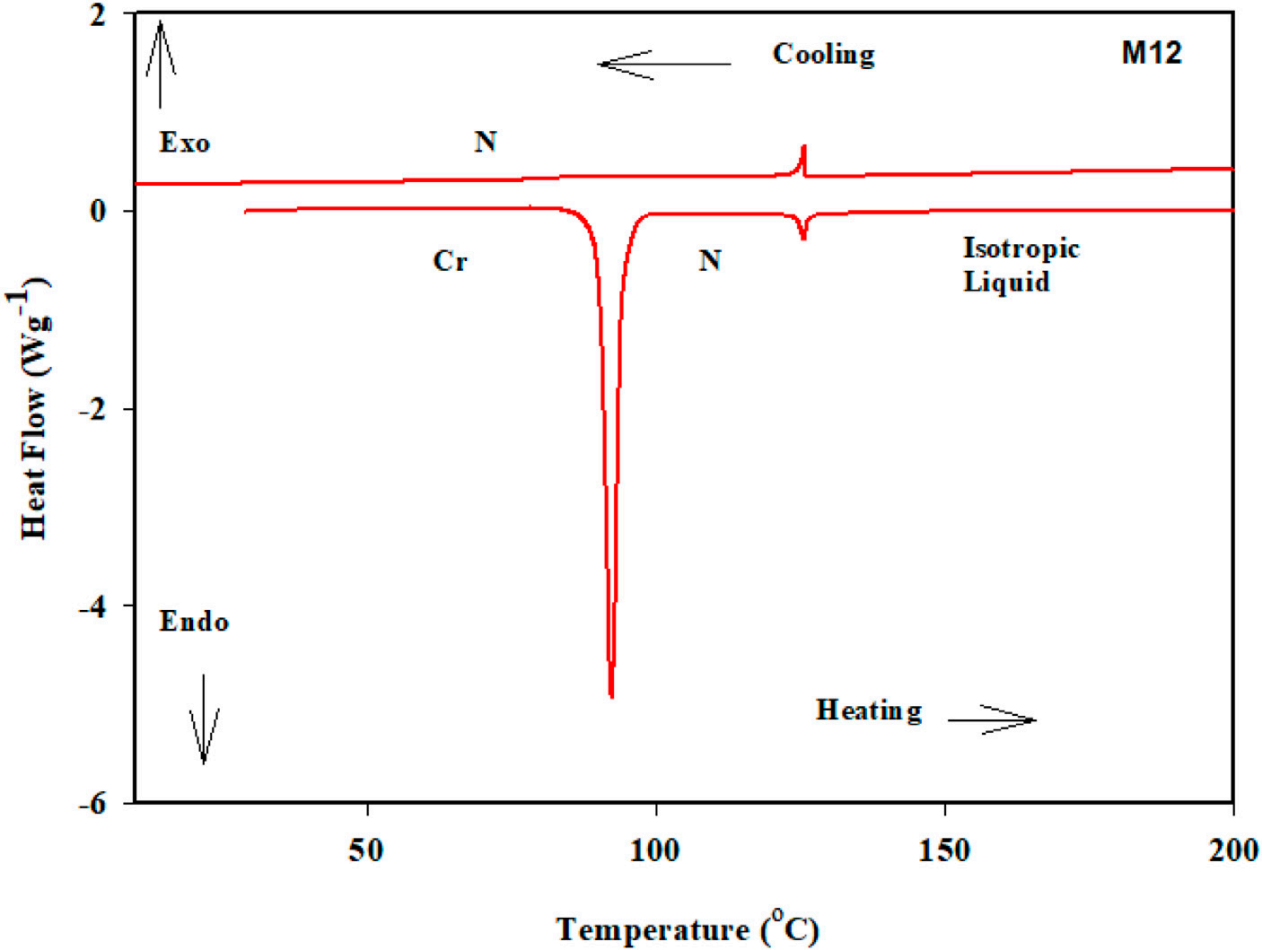
DSC thermograms of **M12** recorded from the second heating and cooling scans at rate ± 10°C/min.

**FIGURE 2:**
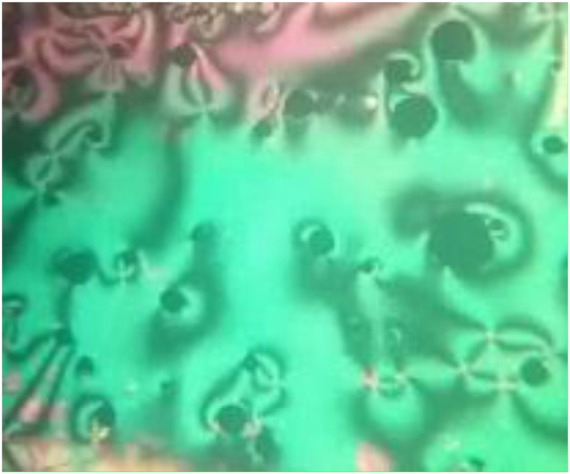
Textures observed under POM for compound **M12** of (a) N phase at 120.0°C upon heating and.

**FIGURE 3:**
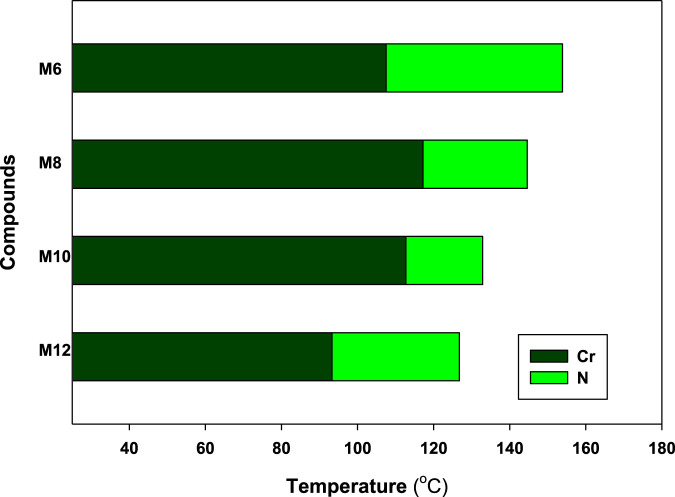
Effect of terminal side alkoxy chain length (n) on mesomorphic characteristics of the **Mn** derivatives of maleic anhydride studied.

DSC and POM measurements have been used to examine the phase transitions and optical properties of the synthesized **Mn** derivatives under investigation. Figure 1 shows an example of a representative DSC thermogram for the proposed compound **M12** after heating and cooling cycles. It is clearly shown that upon heating, the compound **M12** showed two endotherms characteristic of the crystal–N and N–isotropic transitions. While, during the cooling cycle, the compound exhibits nematic phase but its freezing transition temperature is shifted to lower temperature. DSC measurements show two transition peaks that change depending on the **Mn** structural form of the produced materials. Additionally, for all chain lengths (n), the mesomorphic transfers from Cr to N upon heating and N to I upon cooling. According to the attached terminal flexible chain length moiety, which is associated with the mesomorphic interaction, significant endothermic and exothermic peaks were seen, and the cooling cycle corroborated those peaks when the temperature was lowered. The results of the DSC were verified by optical textures under POM. The POM measurements revealed textures which confirmed N mesophases. Figure 2 showed how the **M12** images were derived. Table 1 provides an overview of the mesomorphic transition temperatures and corresponding enthalpies for all the synthesized maleic anhydride derivatives, **Mn**, as determined by DSC analysis. Figure 3 shows their relationships in order to explore how the length of the terminal alkoxy chain (n) affects the mesomorphic behavior of produced compounds. Table 1 and Figure 3 showed that the mesomorphism of all synthesized members of the maleic anhydride derivatives **Mn**, as well as their high mesomorphic thermal stability and good mesophase range, are reliant on the length of their terminal flexible chains. Additionally, all investigated molecules of **Mn** series have a pure N phase and are enantiotropic. Additionally, Table 1 and Figure 3 show that the melting point of compounds varies arbitrarily with chain length (n). The member with the shortest terminal length (**M6**) has the highest nematic thermal stability and enantiotropic N phase at temperatures of 153.9 and 46.4°C, respectively. While the range and nematic stability of the compound **M8** are around 144.6 and 27.4°C, respectively. Enantiotropic N mesophase with nematogenic stability and a range of nearly 132.9 and 20.2°C is also present in the **M10** derivative. Finally, compared to **M8** and **M10**, the derivative with the greatest chain length (**M12**) has a higher mesomorphic range (33.5 C) and weaker thermal N stability (126.8°C). In general, the electronic properties of the terminal substituents have a significant influence on the molecular architecture, polarizability, and dipole moment of the proposed materials. Additionally, an increase in the polarity and/or polarizability of the molecular mesogenic cores has an impact on the mesomorphic nature. The examined homologue’s mesomorphic range expanded in the following order: **M6** > **M12** > **M8** > **M10**. How molecular-molecular interactions affect the mesomorphic behavior of rod-like molecules depends on the geometrical form of the polar terminal groups and the heterocyclic moieties in the molecule (Nafee et al., 2020b). The present prepared group exhibited purely N mesophases, and the N phase stability decreased with n. The interaction between the mesogenic units was reduced as the chain length increased, lowering the N-I transition temperature. The observations of mesomorphic qualities are the result of the sharing of these factors to various degrees. The kind of the observed mesophase is mostly determined by the end-to-end aggregation caused by the oxygen of the alkoxy chain, the ester carbonyl moiety, and the side-by-side cohesive interactions between molecules (Gray, 1962; Luckhurst and Gray, 1979). In general, mesophase stability is enhanced by increasing the polarizability and/or polarity of the entire mesogenic component of the molecule. Although the rod-shaped molecule is less rigid, the increased anisotropic features improve mesophase stability (Yelamagg et al., 2007; Pradhan et al., 2017; Nayak et al., 2019; Sunil et al., 2020). An increase in the alkoxy terminal chain is also projected to reduce stability by diluting the mesogenic core.

**TABLE 1:** Phase transition temperatures, ^o^C (enthalpy of transition ΔH, kJ/mole), mesomorphic range (ΔT, ^o^C) and the normalized entropy of transition, ΔS/R, for present series Fn.

Comp	*T*_Cr-N_	ΔH _Cr-N_	*T*_N-I_	ΔH _N-I_	*ΔT*_N_	*ΔS*_N-I_/R
**M6**	107.5	51.60	153.9	2.76	46.40	0.78
**M8**	117.2	47.82	144.6	2.64	27.40	0.76
**M10**	112.7	41.93	132.9	2.10	20.20	0.62
**M12**	93.3	44.10	126.8	2.15	33.50	0.65

Cr-N, solid—nematic transition.

N-I = nematic—isotropic liquid transition.

R is the universal gas constant.

### 2.4 Entropy changes

Entropy is a crucial factor that must be taken into account while engineering phase transition temperatures. As a result, it is essential to concentrate more on thermodynamic factors in order to create alternative plans for lowering melting and clearing temperatures to levels that complex mesogens can tolerate. Since G = 0, phase transitions are often order-disorder transitions for which H = TS. As a result, the transition entropies can be obtained from the estimated transition enthalpies obtained using differential scanning calorimetry. These are undoubtedly produced as a result of interaction energy attenuation.

Sorai and Saito observed, The alkyl chain can acquire a substantial amount of entropy during disordering, which changes the relative thermodynamic stability of aggregated states (Sorai and Saito, 2003). These chains are incredibly mobile in liquid crystals and act as a sort of solvent. They also explained how the type of phase affects how ordered aliphatic chains are. For instance, the chains have a tendency to be more ordered in lamellar phases compared to cubic bicontinuous phases, whereas the mesogen cores are more disordered in lamellar phases and ordered in cubic phases. Entropy from the chain reservoir can therefore be transported to the core in the scenario where cubic bicontinuous phases change into a lamellar phase. In general, phase transition temperatures can be engineered using the substantial entropy change that results from increasingly ordered chains sequentially increasing entropy until they reach their full degree of freedom in the isotropic phase. Low melting temperatures were ensured by the significant entropy changes at the crystal-liquid crystal transitions of various kinds of mesogens.

Table1 lists the normalized transition entropy changes, or ΔS_N-I_/R, of the currently studied homologues (**Mn**). The results demonstrated that extremely modest entropy variations were found, which were primarily dependent on the type of terminal substituents and msogenic cores. Because of their decreased anisotropy as a result of their molecular geometry and molecular biaxiality, the observed modest values can be explained (Yeap et al., 2011; Henderson et al., 2005; Yeap et al., 2015; Attard et al.). The creation of the mesophase and the molecular organization depend on the molecular orientation, which is influenced by the induction, conjugation forces, particular dipolar contacts, and - stacking interactions (Yeap et al., 2011; Henderson et al., 2005; Yeap et al., 2015; Attard et al.). An increase in dipolar ordering and a decrease in dipolar entropy arise from applying an electrical field to a polar insulator. The dielectric material will return to a less ordered dipolar state once the field is withdrawn, increasing the dipolar entropy (Shanker and Yelamaggad, 2011). In addition, it is more likely to realize a high the electrocaloric effect if the dipolar materials are operated near dipolar order-disorder transitions, where a dipolar ordered state can be created from a dipolar disordered state.

### 2.5 Comparative analysis with related derivatives

It is important to compare the examined materials to analogous ones that have been previously reported with another moiety having the furan substitution as terminal moiety (**Fn**) (Alshabanah et al., 2021) in order to understand the effect of the terminal maleic anhydride moiety on the phase behavior of the materials. Across all parallel series, the maximum allowed number of carbon atoms in the terminal chain (n) was 6, 8, or 12. According to recent findings (Alshabanah et al., 2021), the **Fn** series are intrinsically mesomorphic, exhibiting high mesomorphic thermal stability and a decent mesophase range that varies with the terminal flexible chain length. While molecules **F6** and **F10** are enantiotropic and have pure N phases, the longer chain derivative **F12** contains two mesomorphic transitions (SmA and N phases). The dimorphic characteristics of **F12** demonstrate the existence of an enantiotropic N mesophase and a monotropic SmA phase. However, the nematic phases of the maleic anhydride compounds **Mn** cover all side chain lengths. It is evident that the kind of LC phase has a substantial influence on the terminal replacement. Because only N phases were seen in the maleic anhydride derivatives, the mesomorphic properties were changed when the terminal side by the furan moiety, and the SmA phase developed for lengthy terminal side chain members.

## 3 Experimental

### 3.1 Synthesis

The process used to create the titled Mn compounds involves two sequential steps (Scheme 1):

#### 3.1.1 Synthesis of 1-(4-hydroxyphenyl)-1*H*-pyrrole-2,5-dione, 3

P-Aminophenol (1.637 g, 0.015 mol) and maleic anhydride (1.471 g, 0.015 mol) were separately dissolved in 5 mL of DMF, resulting in solutions A and B, respectively. Solution B was slowly added to solution A, forming solution C. Solution C was then stirred at 20°C in a water bath for 2 h. Next, P_2_O_5_ (1.2 g) was dissolved in a mixture of 1 mL of H_2_SO_4_ and 7 mL of DMF. This mixture was gradually added to solution C while stirring, and the entire solution was further stirred for 2 h at 70°C. To cool the mixture, it was placed in an ice bath and then poured into cold water. Upon precipitation, a solid formed, which was subsequently filtered, washed with distilled water, and finally recrystallized from 2-propanol. The resulting crystals were dried in a vacuum oven at 65°C for 24 h. Yield: 84%; m. p. 184°C–186°C (Lit. m. p. 182°C–184°C (Mohammed and Mustapha, 2010b)).

#### 3.1.2 General procedure for synthesis of 4-(2,5-dioxo-2,5-dihydro-1*H*-pyrrol-1-yl)phenyl 4-(alkoxy)benzoates, Mn

A mixture consisting of 1-(4-hydroxyphenyl)-1H-pyrrole-2,5-dione **3** (1.89 g, 10 mmol) and the appropriate derivatives of 4-alkoxy benzoic acid (10 mmol for each) was prepared in dry methylene chloride (25 mL). To this mixture, N, N′-dicyclohexylcarbodiimide (DCC, 10 mmol) and a small amount of 4-dimethylaminopyridine (DMAP) catalyst were added. The reaction mixture was then stirred continuously at room temperature for 72 h. Afterward, the solid that formed was filtered out, and the solution was evaporated. The resulting solid residue was purified by recrystallization from ethanol, resulting in the production of products with high purity as confirmed by thin-layer chromatography (TLC). TLC sheets coated with silica gel (E Merck) and CH_2_Cl_2/_CH_3_OH (9:1) as the eluent were used, and only one spot was detected under a UV lamp. The structures assigned to the compounds were confirmed by 1H-NMR and elemental analyses, which showed consistent results. The ^1^H-NMR data revealed the expected ratios of integrated aliphatic to aromatic protons for all investigated compounds. The physical data for the products, denoted as **Mn**, are listed below:

##### 3.1.2.1 4-(2,5-Dioxo-2,5-dihydro-1H-pyrrol-1-yl)phenyl 4-(hexyloxy)benzoate M6

Yield: 91.0%; mp 108°C, ^1^H-NMR (500 MHz, CDCl_3_): *δ*/ppm: 0.87–0.91 (t, 3H, CH_3_), 1.29–1.43 (m, 6H, CH_3_(CH_2_)_3_CH_2_CH_2_O-), 1.72–1.74 (m, 2H, CH_3_(CH_2_)_3_CH_2_CH_2_O-), 3.89–3.95 (t, 2H, CH_3_(CH_2_)_3_CH_2_CH_2_O-), 7.11–7.03 (d, 2H, Ar−H), 7.30–7.38 (m, 4H, Ar−H), 7.79 (s, 2H, Pyrrole-H), 8.10–8.16 (d, 2H, Ar−H). ^13^C-NMR (125 MHz, CDCl_3_): *δ*/ppm: 14.1 (CH_3_), 26.0, 29.3, 29.6, 31.9, 68.3 (CH_2_), 121.5, 121.8, 122.4, 132.3, 132.4, 134.9, 143.0, 149.2, 159.6 (Ar-C and C=N), 163.5, 165.0 (C=O); IR (KBr): *v*_cm-1_ 3041 (=C-H), 2928 (-C-H),1686, 1729 (2 C=O), 1605 (C=N), 1571 (C=C); MS, *m/z* (%) 393 (M^+^, 12), 307 (100), 292 (71), 231 (63), 189 (38), 162 (81), 86 (49), 76 (71). Anal. Calcd. for C_23_H_23_NO_5_ (393.44): C, 70.21; H, 5.89; N, 3.56. Found: C, 70.04; H, 5.73; N, 3.39%.

##### 3.1.2.2 4-(2,5-Dioxo-2,5-dihydro-1*H*-pyrrol-1-yl)phenyl 4-(octyloxy)benzoate M8

Yield: 89.0%; mp 117°C, ^1^H-NMR (500 MHz, CDCl_3_): *δ*/ppm: 0.86–0.90 (t, 3H, CH_3_), 1.28–1.44 (m, 10H, CH_3_(CH_2_)_5_CH_2_CH_2_O-), 1.78–1.80 (m, 2H, CH_3_(CH_2_)_5_CH_2_CH_2_O-), 3.88–3.91 (t, 2H, CH_3_(CH_2_)_5_CH_2_CH_2_O-), 7.08–7.14 (d, 2H, Ar−H), 7.31–7.49 (m, 4H, Ar−H), 7.81 (s, 2H, Pyrrole-H), 8.11–8.13 (d, 2H, Ar−H); ^13^C-NMR (125 MHz, CDCl_3_): *δ*/ppm: 14.0 (CH_3_), 22.5, 25.3, 25.7, 29.1, 29.3, 29.6, 29.7, 29.8, 29.9, 31.7, 68.4 (CH_2_), 121.5, 121.9, 122.2, 123.0, 130.7, 133.8, 142.6, 148.9, 159.1 (Ar-C and C=N), 163.3, 164.8 (C=O); IR (KBr): *v*_cm-1_ 3037 (=C-H), 2923 (-C-H), 1683, 1726 (2 C=O), 1601 (C=N), 1568 (C=C); MS, *m/z* (%) 421 (M^+^, 25), 307 (100), 248 (52), 173 (80), 162 (38), 97 (53), 76 (66). Anal. Calcd. for C_25_H_27_NO_5_ (421.49): C, 71.24; H, 6.46; N, 3.32. Found: C, 71.04; H, 6.28; N, 3.23%.

##### 3.1.2.3 4-(2,5-Dioxo-2,5-dihydro-1*H*-pyrrol-1-yl)phenyl 4-(decyloxy)benzoate M10

Yield: 92.0%; mp 113°C, ^1^H-NMR (500 MHz, CDCl_3_): *δ*/ppm: 0.79–0.81 (t, 3H, CH_3_), 1.21–1.41 (m, 14H, CH_3_(CH_2_)_7_CH_2_CH_2_O-), 1.71–1.75 (m, 2H, CH_3_(CH_2_)_7_CH_2_CH_2_O-), 3.81–3.96 (t, 2H, CH_3_(CH_2_)_7_CH_2_CH_2_O-), 7.13–7.20 (d, 2H, Ar−H), 7.29–7.42 (m, 4H, Ar−H), 7.79 (s, 2H, Pyrrole-H), 8.10–8.13 (d, 2H, Ar−H). ^13^C-NMR (125 MHz, CDCl_3_): *δ*/ppm: 14.1 (CH_3_), 22.7, 25.9, 29.1, 29.3, 29.5, 29.6, 29.7, 31.9, 68.3 (CH_2_), 121.1, 121.4, 121.8, 122.4, 132.3, 134.9, 143.0, 149.2, 159.6 (Ar-C and C=N), 163.5, 164.2 (C=O); IR (KBr): *v*_cm-1_ 3039 (=C-H), 2920 (-C-H), 1680, 1722 (2 C=O), 1603 (C=N), 1568 (C=C); MS, *m/z* (%) 449 (M^+^, 21), 307 (100), 276 (47), 173 (63), 142 (55), 76 (61). Anal. Calcd. for C_27_H_31_NO_5_ (449.55): C, 72.14; H, 6.95; N, 3.12. Found: C, 72.03; H, 6.79; N, 3.00%.

##### 3.1.2.4 4-(2,5-Dioxo-2,5-dihydro-1*H*-pyrrol-1-yl)phenyl 4-(dodecyloxy)benzoate M12

Yield: 90%; mp 93°C, ^1^H-NMR (500 MHz, CDCl_3_): *δ*/ppm: 0.73–0.75 (t, 3H, CH_3_), 1.10–1.35 (m, 18H, CH_3_(CH_2_)_9_ CH_2_CH_2_O-), 1.59–1.69 (m, 2H, CH_3_(CH_2_)_9_CH_2_CH_2_O-), 3.85–3.92 (t, 2H, CH_3_(CH_2_)_9_CH_2_CH_2_O-), 7.10–7.13 (d, 2H, Ar−H), 7.40–7.48 (m, 4H, Ar−H), 7.81 (s, 2H, Pyrrole-H), 8.13–8.17 (d, 2H, Ar−H); ^13^C-NMR (125 MHz, CDCl_3_): *δ*/ppm: 14.1 (CH_3_), 22.7, 25.6, 25.9, 29.3, 29.5, 29.6, 29.7, 29.7, 29.8, 31.9, 68.3 (CH_2_), 121.1, 121.4, 122.4, 123.2, 132.2, 134.9, 143.0, 149.4, 159.6 (Ar-C and C=N), 163.5, 165.0 (C=O); IR (KBr): *v*_cm-1_ 3025 (=C-H), 2917 (-C-H), 1681, 1719 (2 C=O), 1600 (C=N), 1545 (C=C); MS, *m/z* (%) 477 (M^+^, 18), 307 (100), 247 (52), 173 (46), 97 (83), 76 (51). Anal. Calcd. for C_29_H_35_NO_5_ (477.60): C, 72.93; H, 7.39; N, 2.93. Found: C, 72.82; H, 7.31; N, 2.85%.

## 4 Conclusion

In this study, 4-(2,5-dioxo-2,5-dihydro-1*H*-pyrrol-1-yl)phenyl 4-(alkoxy)benzoates (**Mn**), a novel optical liquid crystalline homologue based on the maleic anhydride molecule, was created and mesomorphically examined using DSC and POM. Mesomorphic and optical characterizations showed that all of the studied set’s produced compounds are monomorphic and display enantiotropic liquid crystalline N mesophases. A wide range of N stability has also been provided by the addition of a heterocyclic moiety to the molecular structure. The entropy increases associated with the N-isotropic transitions are minor in magnitude and follow an erratic trend that is independent of the terminal alkoxy chain length (n). This may be due to the comparatively high clearing temperature values and the smallest promotion of molecular biaxiality by the ester linkage group. Finally, it was established that the substitution under investigation causes the formation of nematic phases by contrasting the materials under investigation with their related furan compounds described in the literature.

## Data Availability

The original contributions presented in the study are included in the article/Supplementary Material, further inquiries can be directed to the corresponding author.
