# Supplementary material for: Novel maleic anhydride derivatives: liquid crystalline materials with enhanced mesomorphic and optical characteristics
Source: Front Chem. 2023 Nov 9;11:1287883. doi: 10.3389/fchem.2023.1287883 (PMC10665853; doi:10.3389/fchem.2023.1287883)
Supplement: Supplementary file 1 [file DataSheet1.docx]

**Supplementary data**

**Novel Maleic Anhydride Derivatives: Liquid Crystalline Materials with Enhanced Mesomorphic and Optical Characteristics**

**Hoda A. Ahmed ^1,2,*^, Tariq Z. Abolibda^3^, Yasser A. M. Ismail ^4^, Abdullah Almohammedi ^4^, K. A. Aly ^5,6^, Mohamed S. Ibrahim ^3^, Sobhi M. Gomha ^3^**

^1^Department of Chemistry, Faculty of Science, Cairo University, Cairo 12613, Egypt

^2^Chemistry Department, College of Sciences, Taibah University, Yanbu 30799, Saudi Arabia

^3^Department of Chemistry, Faculty of Science, Islamic University of Madinah, Madinah 42351, Saudi Arabia

^4^Department of Physics, Faculty of Science, Islamic University of Madinah, Saudi Arabia

^5^Department of Physics, Collage of Science and Arts, Jeddah University, Saudi Arabia

^6^Department of Physics, Faculty of Science, Al-Azhar University, Asyut 71121, Egypt

* Correspondence: [ahoda@sci.cu.edu.eg](mailto:ahoda@sci.cu.edu.eg) (H.A.A.)

1. ***Materials***

4-Aminophenol, maleic anhydride, 4-hexyloxybenzoic acid, 4-octyloxybenzoic acid, 4-decyloxybenzoic acid, 4-dodecyloxybenzoic acid and were purchased from Sigma Aldrich (Germany). 4-dimethylaminopyridine (DMAP) were purchased from Aldrich (Wisconsin, USA), dichloromethane, ethanol and *N,N'*-dicyclohexylcarbodiimide (DCC),.

1. ***Characterizations***

Melting points were measured using an Electrothermal Gallenkamp digital melting point apparatus and are reported uncorrected. ^1^H-NMR spectra were recorded using deuterated chloroform (CDCl_3_) solution on a Varian Mercury VX-500 MHz spectrometer and ^13^C-NMR spectra were recorded at 125 MHz. Chemical shifts are quoted in *δ* and were reported related to that of solvents. IR spectra were recorded in potassium bromide discs using PyeՍnicam SP-1000 spectrometer. Mass spectra were recorded using a Shimadzu GCMS-Qp-2010 Plus mass spectrometer (Tokyo, Japan) operating at 70 eV. Elemental analyses were carried out by the Microanalytical Centre of Cairo University, Giza, Egypt.

Differential Scanning Calorimeter, TA instrument Co. Q20 (DSC; USA), was used for calorimetric measurements. The melting point and enthalpy of indium and lead were used for DSC calibration. Aluminum pans and (2–3 mg) sample amounts were used for DSC measurements investigation. (30 ml/min) nitrogen gas inert atmosphere and 10°C/min heating rate were selected for all measurements and all transition were recorded from the second heating scan. The types of the mesophase texture were identified by a standard polarized optical microscope (POM, Wild, Germany) with Mettler FP82HT hot stage and the temperature controller was attached as thermocouple for temperature measurements. All recorded values were made twice and the results have accuracy ± 0.2°C for transition temperature.


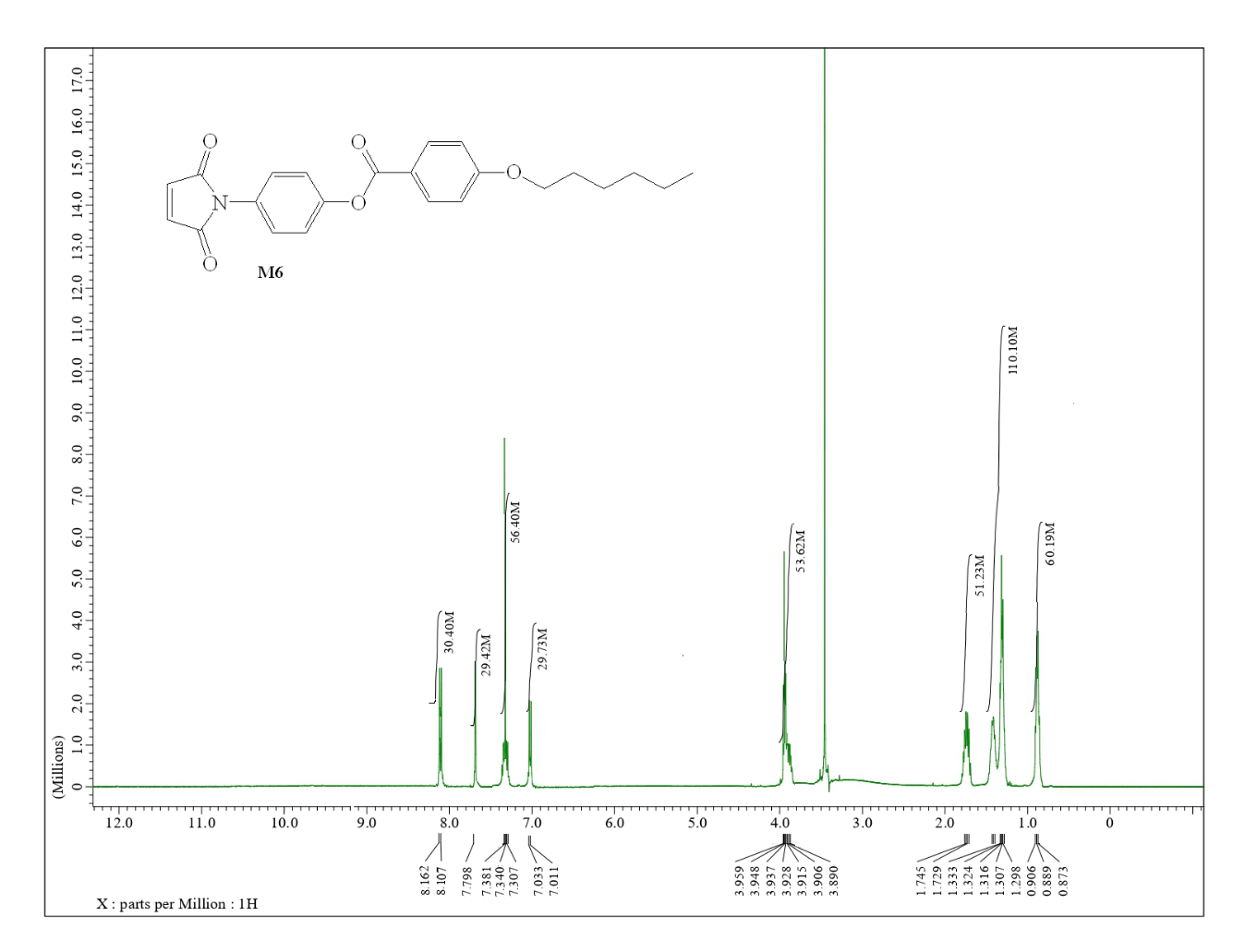


^1^H-NMR spectra of compound M6

**
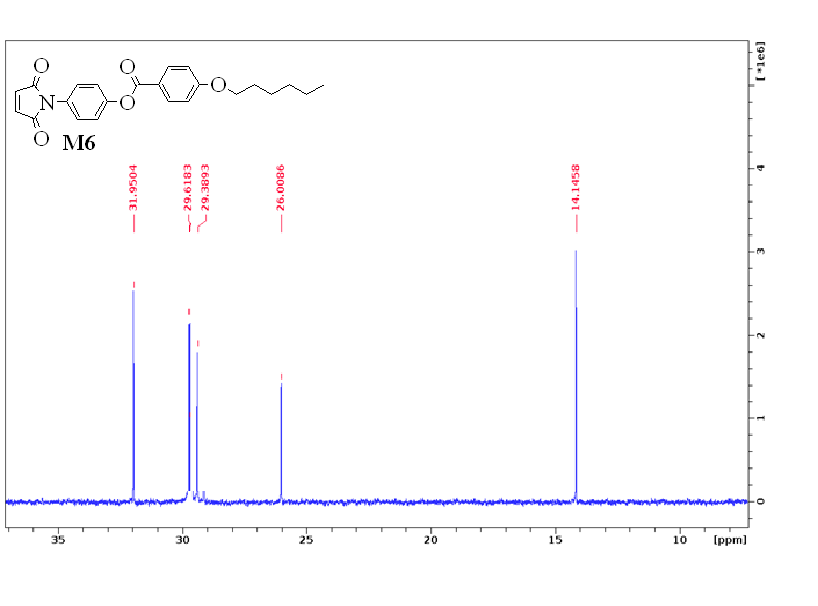
**

^13^C-NMR spectra of compound M6 (1)


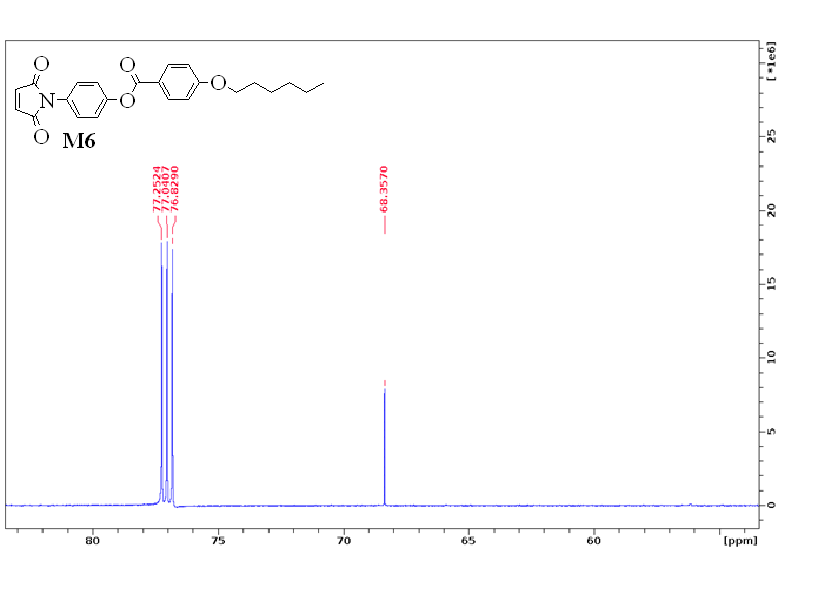


^13^C-NMR spectra of compound M6 (2)


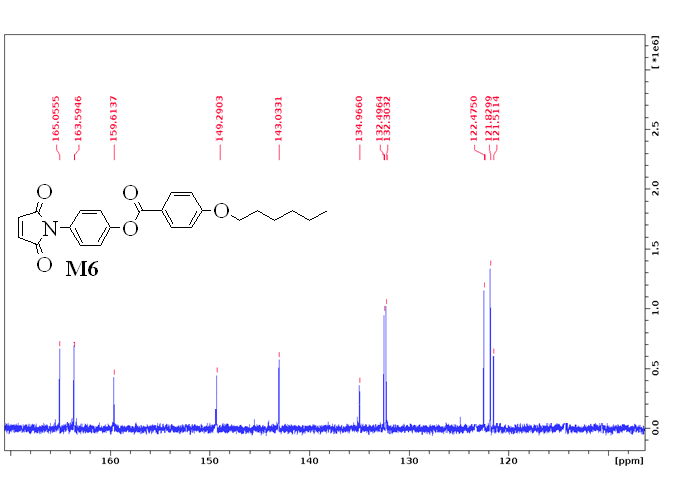


^13^C-NMR spectra of compound M6 (3)


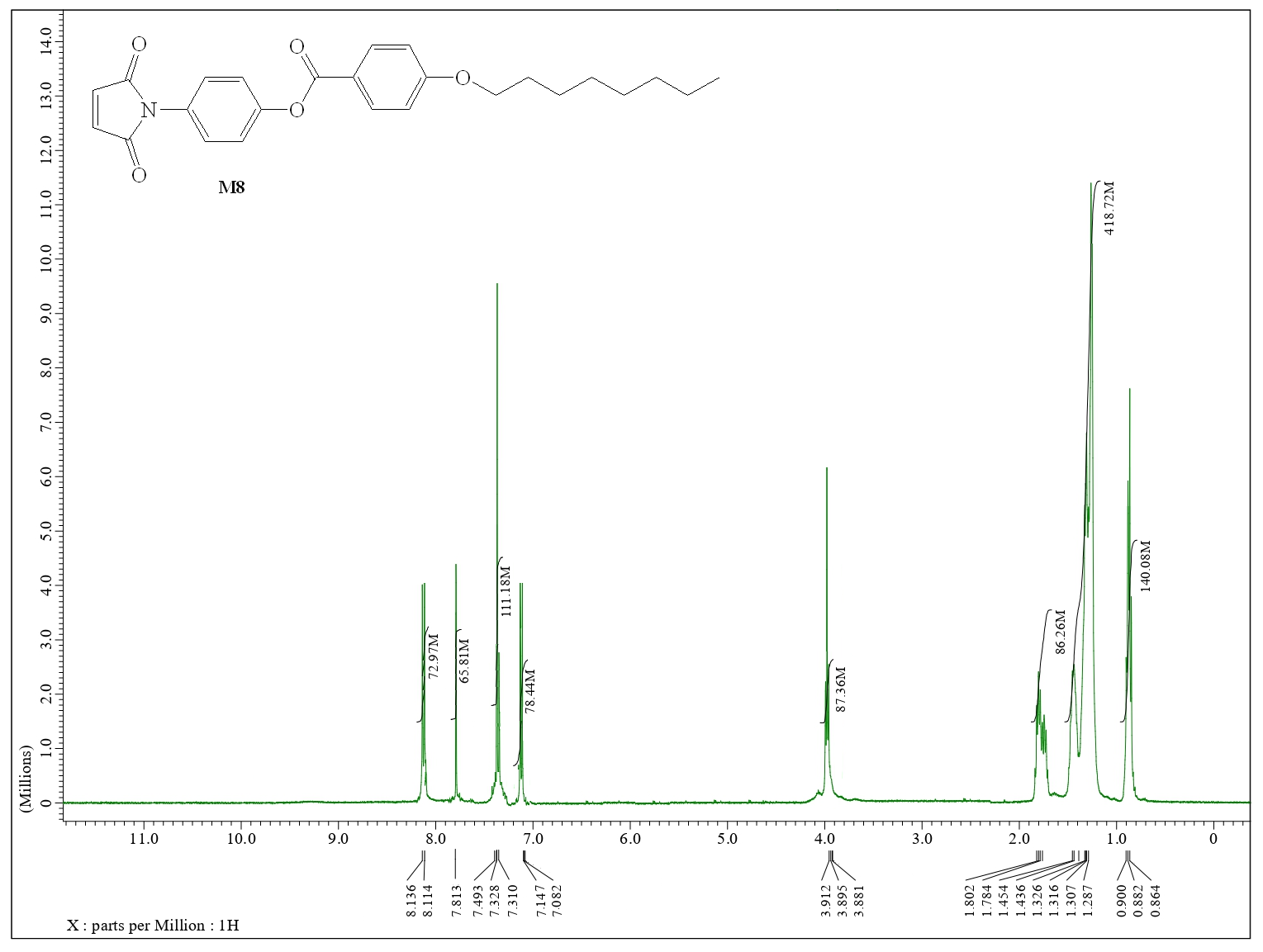


^1^H-NMR spectra of compound M8


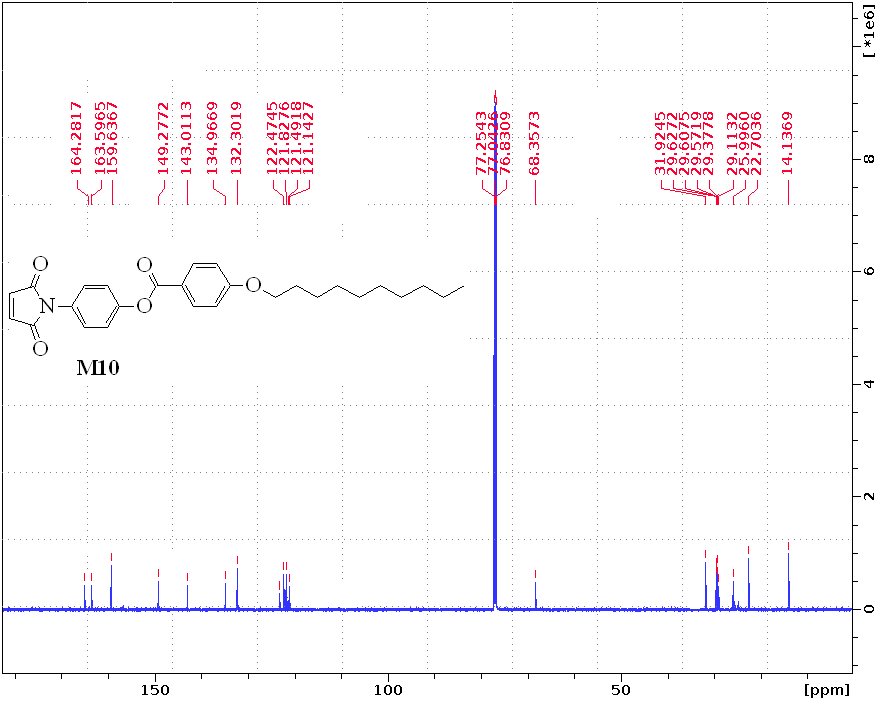


^13^C-NMR spectra of compound M10


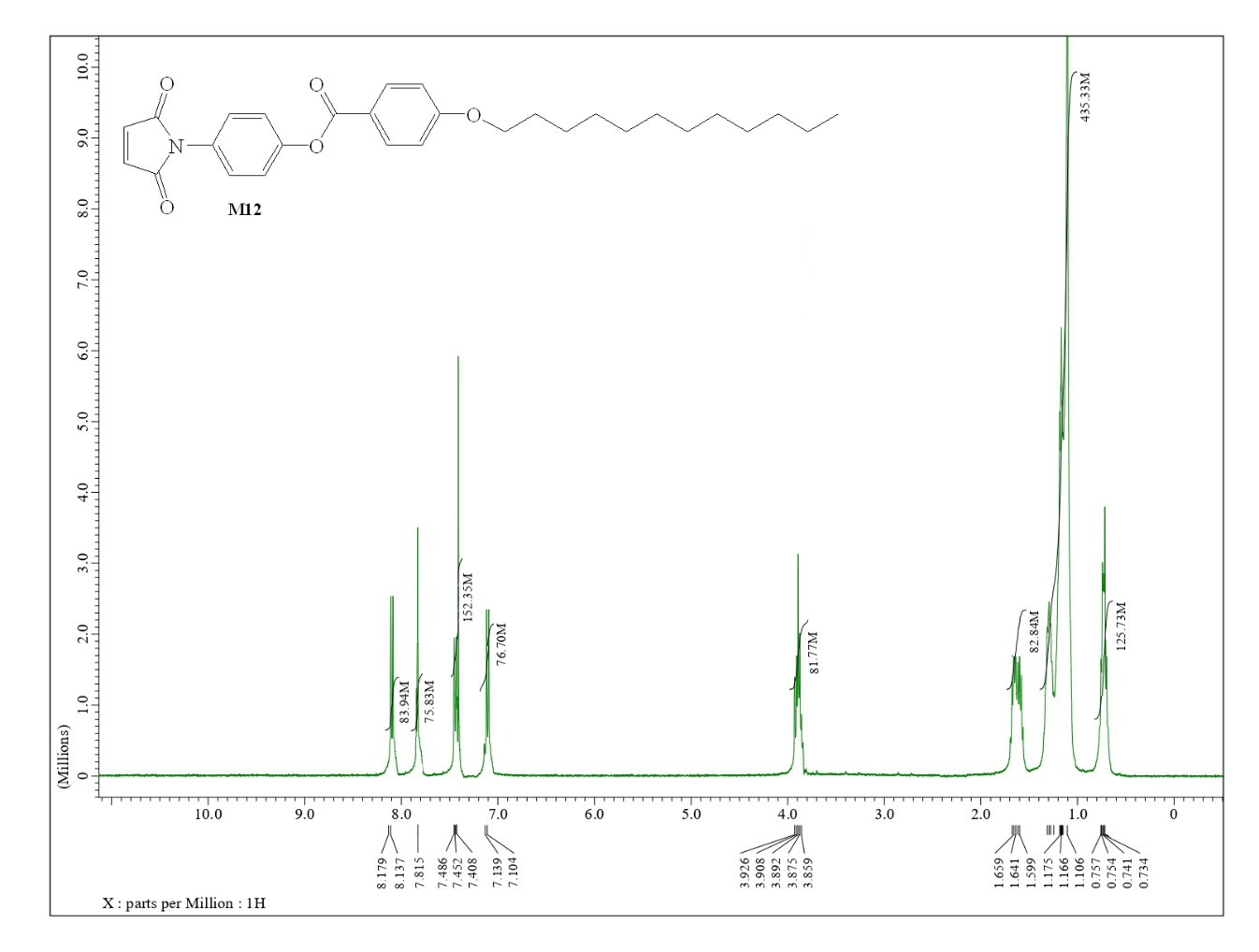


^1^H-NMR spectra of compound M12


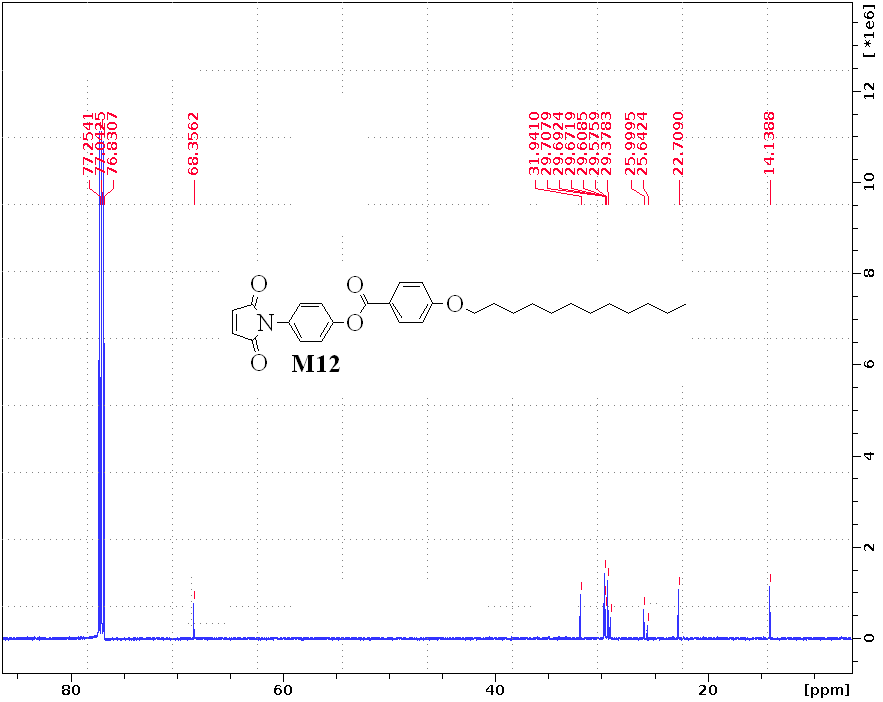


^13^C-NMR spectra of compound M12 (1)


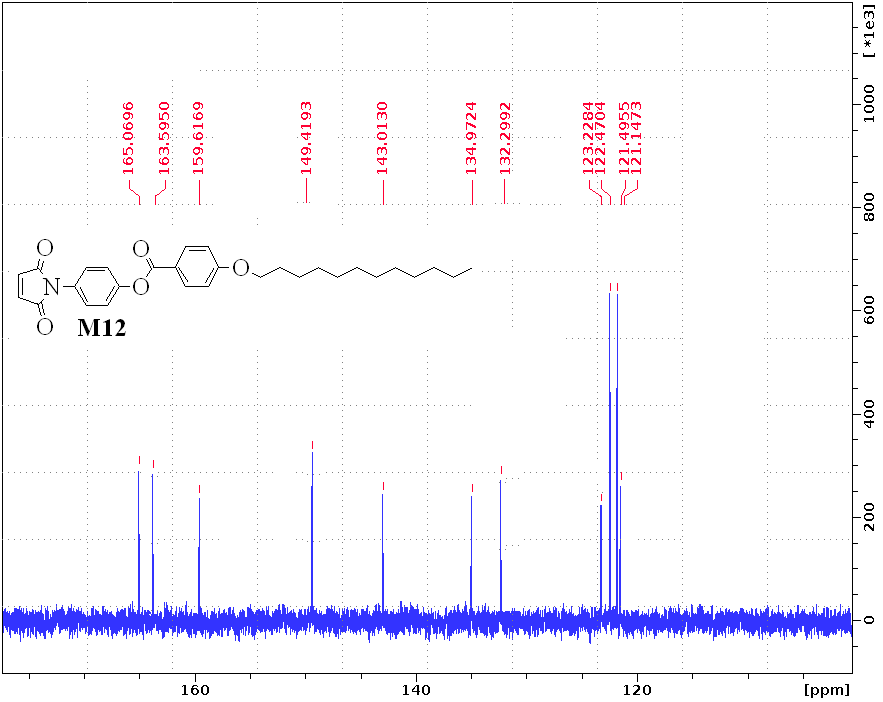


^13^C-NMR spectra of compound M12 (2)
